# Xenoantigenicity of porcine decellularized valves

**DOI:** 10.1186/s13019-017-0621-5

**Published:** 2017-07-17

**Authors:** Meghana R. K. Helder, Nicholas J. Stoyles, Brandon J. Tefft, Ryan S. Hennessy, Rebecca R. C. Hennessy, Roy Dyer, Tyra Witt, Robert D. Simari, Amir Lerman

**Affiliations:** 1 Department of Cardiovascular Surgery, Mayo Clinic, Rochester, MN USA; 2 Department of Cardiovascular Diseases, Mayo Clinic, 200 First St SW, Rochester, MN 55905 USA; 3 Divisions of Immunochemical Core Lab, Mayo Clinic, Rochester, MN USA

**Keywords:** Valve deterioration, Valve durability, Immune response

## Abstract

**Background:**

The xenoantigenicity of porcine bioprosthetic valves is implicated as an etiology leading to calcification and subsequent valve failure. Decellularization of porcine valves theoretically could erase the antigenicity of the tissue leading to more durable prosthetic valves, but the effectiveness of decellularization protocols in regard to completely removing antigens has yet to be verified. Our hypothesis was that decellularization would remove the more abundant α-gal antigens but not remove all the non α-gal antigens, which could mount a response.

**Methods:**

Porcine aortic valves were decellularized with 1% sodium dodecyl sulfate for 4 days. Decellularized cusps were evaluated for α-gal epitopes by ELISA. To test for non α-gal antigens, valves were implanted into sheep. Serum was obtained from the sheep preoperatively and 1 week, 1 month, and 2 months postoperatively. This serum was utilized for anti-porcine antibody staining and for quantification of anti-pig IgM and IgG antibodies and complement.

**Results:**

Decellularized porcine cusps had 2.8 ± 2.0% relative α-gal epitope as compared to fresh porcine aortic valve cusps and was not statistically significantly different (*p* = 0.4) from the human aortic valve cusp which had a 2.0 ± 0.4% relative concentration. Anti-pig IgM and IgG increased postoperatively from baseline levels. Preoperatively anti-pig IgM was 27.7 ± 1.7 μg/mL and it increased to 71.9 ± 12.1 μg/mL average of all time points postoperatively (*p* = 0.04). Preoperatively anti-pig IgG in sheep serum was 44.9 ± 1.5 μg/mL and it increased to 72.6 ± 6.0 μg/mL average of all time points postoperatively (*p* = 0.01). There was a statistically significant difference (*p* = 0.00007) in the serum C1q concentration before valve implantation (2.5 ± 0.2 IU/mL) and at averaged time points after valve implantation (5.3 ± 0.3 IU/mL).

**Conclusions:**

Decellularization with 1% sodium dodecyl sulfate does not fully eliminate non α-gal antigens; however, significant reduction in α-gal presence on decellularized cusps was observed. Clinical implications of the non α-gal antigenic response are yet to be determined. As such, evaluation of any novel decellularized xenografts must include rigorous antigen testing prior to human trials.

**Electronic supplementary material:**

The online version of this article (doi:10.1186/s13019-017-0621-5) contains supplementary material, which is available to authorized users.

## Background

The scarcity of aortic valve allografts has led to a large amount of research utilizing porcine aortic valves as a valve replacement option [1]. Commercial porcine bioprosthetic valves are widely used and are arguably a hemodynamically superior option for aortic valve replacement as compared to mechanical valves, and avoid the need for chronic anticoagulation. However, all commercial porcine bioprosthetic valves are currently glutaraldehyde fixed and tend to fail secondary to dystrophic calcification in as early as 5 years post-implantation [2]. Glutaraldehyde fixation is meant to “hide” antigens from the host, but significant antigenicity remains nevertheless. In fact, antibodies to xenoantigens have been identified in patients after implantation of porcine bioprostheses [3]. Failure of porcine valves has been attributed to both glutaraldehyde fixation [4] and xenogeneic antigen-triggered immune response evident even with fixed valves [5].

The host immune response to xenogeneic valves is not well understood, but likely involves both the innate and acquired immune systems. The antibody response is primarily triggered by the cell surface epitope galactose-alpha 1–3-galactose (α-gal), which is an antigen found on porcine valves. Antibodies are likely also formed to other non α-gal antigens, as immune response still occurs to tissues derived from α-gal knockout animals due to the presence of unknown xenogeneic antigens [6]. The initial antibody response triggers a downstream inflammatory reaction. For example, T cell infiltration has been seen in response to porcine small intestine. This likely leads to a production of cytokines and a pro-inflammatory milieu [7].

Decellularization of porcine aortic valves has been touted to avoid the problems seen with commercial porcine valves as there would be no need for glutaraldehyde fixation and the tissue could be repopulated with host cells. Also, theoretically, decellularization would remove all cellular material and DNA, reducing antigenicity if not completely eliminating it. There have been conflicting effects of decellularization on the xenogeneic antigen α-gal [8, 9]. Other in vitro studies have looked at immune response pathways when decellularized porcine valves are in contact with human substrate. When decellularized valves contact human blood, the classical complement pathway is activated [10]. Other in vitro studies have shown cytokine release in cell proliferation assays after exposure to decellularized porcine valves [11].

We have previously published our experience with implanting decellularized porcine valves into sheep [12]. The increasing gradients that we observed across our implanted valve on echocardiography prompted us to explore the immune response in these sheep as a reason for valve failure. We hypothesized that non α-gal antigens still existed after decellularization of porcine tissue and aimed to study the immune response in vivo utilizing a sheep model, given that sheep do not produce α-gal antibodies.

## Methods

Below are the concise methods for each step. Please see Additional file 1: Methods supplement for more detail.

### Heart valve processing

Aortic valves, inclusive of both the anterior leaflet of the mitral valve and the root, were dissected from porcine hearts obtained from a local slaughterhouse. Hearts were kept on ice and valves were dissected within 2 h of harvest in order to minimize postmortem degradation of the extracellular matrix. Immediately after dissection, the valves were decellularized. Decellularization began with 4 days of 1% sodium dodecyl sulfate (SDS) and was followed with 2 days of Tris buffer, DNAse and MgCl_2_, 2 days of PBS washing, 2 days of 0.1% peracetic acid, and 21 days of PBS washing in that order. In our preliminary studies, we determined 4 days of SDS solubilization is needed to remove >98.5% of DNA content from the valve tissue (data not shown). After decellularization, terminal sterilization was performed with gamma irradiation (1500 Gy or 3000 Gy).

### α-gal epitope ELISA

An ELISA development kit was purchased from KPL (Gaithersburg, MD, product 54–62-18). Human serum albumin (Sigma-Aldrich, St. Louis, MO, product A1653-5G) was used as a blocking agent. 1.5–3 μg of protein extracts of fresh porcine aortic cusps (positive control, *n* = 5), decellularized porcine aortic cusps (*n* = 5), and human aortic cusps (negative control, *n* = 3) were used to coat 96-well polystyrene ELISA plates. The primary antibody was the α-gal monoclonal antibody, M86 (Enzo Life Sciences, Inc., product ALX-801-090). The secondary antibody was a goat anti-mouse IgG-HRP. Absorbance was determined at 405 nm and background absorbance was subtracted. Relative concentrations of α-gal epitopes were calculated based on fresh porcine cusp defined as 100%.

### Animals and operative model

Juvenile sheep (*n* = 3) were 3 to 4 months of age and 30 to 40 kg in mass and underwent a pulmonary valve replacement with decellularized and sterilized porcine aortic valves. All animals received humane care in compliance with the Principles of Laboratory Animal Care formulated by the National Society for Medical Research and the Guide for the Care and Use of Laboratory Animals. The study protocol was approved by the Institutional Animal Care and Use Committee at Mayo Clinic. The operations were performed through a left thoracotomy utilizing the fourth intercostal space to gain exposure. After heparinization, normothermic cardiopulmonary bypass was utilized to empty the heart, but the heart was not arrested. The main pulmonary artery was incised 1 cm above the right ventricular outflow tract and just below the bifurcation of the pulmonary artery. The native pulmonary valve cusps were excised. The decellularized heart valve was interposed in the pulmonary position with running sutures of 4–0 polypropylene proximally and distally. Intercostal nerve blocks were performed with 0.5% bupivacaine and epinephrine mixture prior to closure of the thoracotomy. Ceftiofur 5 mg/kg was given intramuscularly the day before the surgery and was repeated on the 3rd postoperative day. Cefazolin 50 mg/kg was given intravenously 15 mins prior to incision. Animals were kept indoors for the first week postoperatively and given 25 U/kg of Heparin twice a day for 2 days postoperatively. Vital signs were monitored daily for 1 week [12].

### Serum for immune testing

Ten mL of blood was drawn into clot tubes from each sheep (*n* = 3) preoperatively, 1 week postoperatively, 1 month postoperatively, and 2 months postoperatively (with the exception of our first sheep which was sacrificed at 1 month). The blood was centrifuged immediately at 3000 RPM for 10 mins. The top layer was stored at −80 °C for later analysis.

### Anti-pig antibody staining

Fresh frozen blocks of 3000 Gy gamma irradiated (*n* = 4) decellularized porcine aortic valve cusps were cut into 5 μm sections. Each section was incubated with a 1:25 dilution of sheep serum (obtained at different time points as described above) in 4 °C for 18 h. Secondary antibodies were rabbit anti-sheep IgM:FITC (AbD Serotec) and donkey anti-sheep IgG:Alexa Fluor 594 (Life Technologies). ProLong Gold Antifade with DAPI (Life Technologies) was utilized for preservation and counterstaining. Our negative staining controls utilized PBS rather than sheep serum. All images were taken with a confocal microscope [13].

### Anti-pig antibody ELISA

Microtiter wells (Thermo Scientific, Waltham, MA) were coated with 100 μL of a 10 μg/mL extract of decellularized porcine valves. Sheep serum from the 3 sheep implanted with decellularized valves was added to the antigen-coated wells (100 μL of a 25 fold dilution in PBS). A mixture (100 μL of a 3 μg/mL solution of each) of FITC conjugated anti-IgM (Bio-Rad, Hercules, CA) and Alexaflour 568 conjugated anti-IgG (Life Technologies, Grand Island, NY) was added. The fluorescence was measured using the maximum excitation and emission wavelengths for each fluorophore. Purified sheep antibodies from Fitzgerald Industries (Acton, MA), IgG (product 31R-1050) and IgM (product 31C–CH1313), were used to produce standard curves.

### Cytotoxic T-lymphocyte staining

A portion of each cusp of the explanted valves (*n* = 3) was created into frozen sections. Anti-CD8 antibody (Abcam) was the primary antibody. Goat anti-rabbit Alexafluor 488 (Life Technologies) was the secondary antibody. ProLong Gold Antifade with DAPI (Life Technologies) was utilized for preservation and counterstaining. The cusp of the sheep known to have endocarditis on the valve (previously published results (12)) was utilized as a positive control and the other 2 sheep with no evidence of infection were used to determine the result. All images were taken with a confocal microscope.

### Quantification of complement

Sheep C1q ELISA kit was purchased from BioTang and utilized to measure the pre and postoperative sheep serum samples of the 3 sheep implanted with decellularized valves. Samples were diluted 1:40 with the provided sample buffer. Standards and samples were run in technical triplicates and the experiment was repeated twice.

### Statistical analysis

Quantitative variables from ELISA were analyzed using a mixed linear effects model to test the effect of the time points on each concentration. The time point variable was treated as a fixed effect, sheep was treated as a random effect, and the replicate was handled as a random effect nested within sheep and time point. We conducted linear contrasts to test the differences between the post implantation time points with the baseline preoperative value, after inspecting the overall effect of the time point variable. A two-tailed t-test was used to evaluate the difference in the α-gal epitope of decellularized and human aortic cusps. All analyses were conducted using JMP software. Values are listed as mean ± standard error. *P* < 0.05 was considered significant.

## Results

### α-gal epitope ELISA

**Fig. 1:**
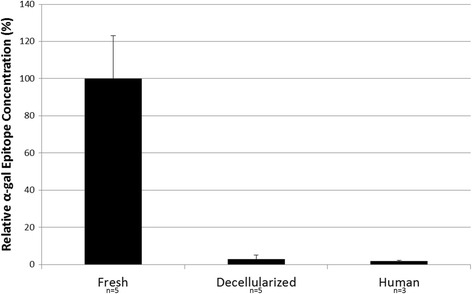
Alpha-gal ELISA assay- Relative concentrations of α-gal epitopes were calculated based on fresh porcine cusp defined as 100%. Decellularized cusps had 2.8 ± 2.0% relative α-gal epitope while human aortic cusps had 2.0 ± 0.4% as compared to fresh porcine aortic valve cusps. The relative concentration of α-gal epitope in the decellularized cusps was not statistically significantly different (*p* = 0.4) from the human aortic cusp

A fresh porcine cusp (*n* = 5) was used as a positive control and had an absorbance of 0.56 ± 0.1. Decellularized cusps (*n* = 5) had an absorbance of 0.02 ± 0.01. The negative control of the human aortic cusp (*n* = 3) had an absorbance of 0.014 ± 0.002. Decellularized cusps had 2.8 ± 2.0% relative α-gal epitope while human aortic cusps had 2.0 ± 0.4% as compared to fresh porcine aortic valve cusps. The relative concentration of α-gal epitope in the decellularized cusps was not statistically significantly different (*p* = 0.4) from the human aortic cusp (Fig. 1).

### Anti-pig antibody staining

**Fig. 2:**
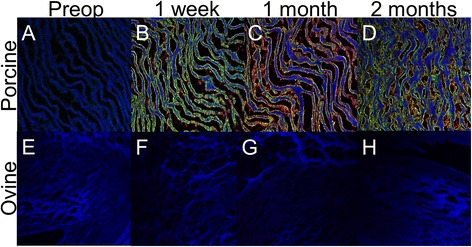
Anti-pig antibody staining- Detection of anti-pig antibodies in sheep serum. **a.** Sheep serum taken preoperatively showed no evidence of anti-pig antibodies and as such there is no green or red fluorescence. The *blue* is auto-fluorescence of decellularized porcine valve with the addition of DAPI. **b.** Sheep serum taken at 1 week postoperatively shows an abundance of anti-pig IgM antibodies (*green*) and evidence of anti-pig IgG antibodies (*red*). The autoflorescence of decellularized porcine valve with the addition of DAPI (*blue*) is also seen. **c-d.** Sheep serum taken at 1 month and 2 months postoperatively shows an abundance of anti-pig IgM (*green*) and IgG (*red*) antibodies. **e-h.** Sheep serum at each time point was incubated with decellularized ovine valves. There is no evidence on any IgM (*green*) or IgG (*red*) antibodies. The addition of DAPI appears as autoflourescene of decellularized ovine valves. This was a negative control to show that antibody binding was specific to porcine tissue

There was no detection of antibodies when sterilized, decellularized porcine cusps were incubated with sheep serum obtained preoperatively. However, at all time points postoperatively, antibodies in sheep serum interacted with the antigens on sterilized, decellularized porcine cusps implicating the presence of anti-porcine antibodies within the serum (Fig. 2). At the 1 week postoperative time point mainly IgM antibodies were seen binding, but by 1 month IgG antibodies were also present. At the 2 month time point, both antibodies were present. Images were consistent among all three sheep serum samples. Sterilized ovine decellularized cusps were utilized as negative control and no binding of antibodies was evident, implying that the antibodies in the sheep serum were xenoantibodies.

### Anti-pig ELISA

**Fig. 3:**
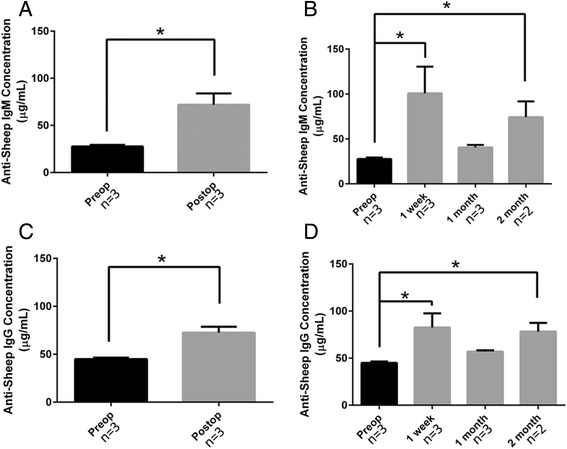
Quantification of anti-pig IgM and IgG antibodies in sheep serum- **a.** There was a statistically significant increase in serum anti-pig IgM antibodies in postoperative time points (71.9 ± 12.1 μg/mL) as compared to before valve implantation (27.7 ± 1.7 μg/mL, *p* = 0.04). **b.** When comparing each postoperative time point separately to the preoperative serum levels, there was a significant increase in serum anti-pig IgM antibodies by 1 week (100.8 ± 29.6 μg/mL, *p* = 0.009). At 1 month, serum IgM concentrations were 40.6 ± 2.8 μg/mL (*p* = 0.2) and at 2 months were 74.3 ± 17.5 μg/mL (*p* = 0.04). **c.** There was a statistically significant increase in serum anti-pig IgG antibodies in postoperative time points (72.6 ± 6.0 μg/mL) as compared to before valve implantation (44.9 ± 1.5 μg/mL, *p* = 0.01). **d.** When comparing each postoperative time point separately to the preoperative serum levels, there was a significant increase in serum anti-pig IgG antibodies by 1 week (82.6 ± 14.9 μg/mL, *p* = 0.002). At 1 month, serum IgG concentrations were 56.9 ± 1.4 μg/mL (*p* = 0.3) and at 2 months were 78.3 ± 9.2 μg/mL (*p* = 0.02)

Both anti-pig IgG and IgM significantly increased after valve implantation (Fig. 3). Preoperatively, anti-pig IgM was 27.7 ± 1.7 μg/mL and it increased to 71.9 ± 12.1 μg/mL average of all time points postoperatively (*p* = 0.04). When examining each postoperative time point separately, there was a significant increase in serum anti-pig IgM antibodies by 1 week (100.8 ± 29.6 μg/mL, *p* = 0.009). There was a drop in serum concentrations towards baseline by 1 month (40.6 ± 2.8 μg/mL, *p* = 0.2) but a rise again at 2 months (74.3 ± 17.5 μg/mL, *p* = 0.04). Preoperatively, anti-pig IgG in sheep serum was 44.9 ± 1.5 μg/mL and it increased to 72.6 ± 6.0 μg/mL average of all time points postoperatively (*p* = 0.01). When examining each postoperative time point separately, there was a significant increase in serum anti-pig IgG antibodies by 1 week (82.6 ± 14.9 μg/mL, *p* = 0.002). There was a drop in serum concentrations towards baseline by 1 month (56.9 ± 1.4 μg/mL, *p* = 0.3) but a rise again at 2 months (78.3 ± 9.2 μg/mL, *p* = 0.02).

### Cytotoxic T-lymphocyte staining

**Fig. 4:**
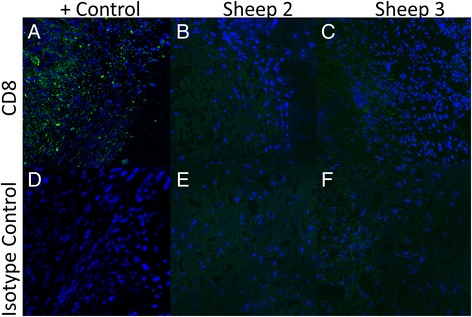
Evaluation of explanted valve tissue for cytotoxic T-cells**- a.** Positive control. Anti-CD8 staining of the explanted valve known to be infected with bacteria and thus would have an expected cytotoxic T-cell population (*green*). Cell nuclei are stained with DAPI (*blue*). **b**-**c.** Representative images of the other two valves explanted from sheep that had no evidence of endocarditis. Cell nuclei (*blue*) are likely from recellularization by the host. There is no evidence of cytotoxic T-cells (*green*). **d**-**f.**) Isotype staining controls of valves explanted from all three sheep. Cell nuclei are present (*blue*) showing recellularization again by host, but there is no evidence of secondary antibody binding (*green*)

CD8 positive cells were present on the positive control of the sheep valve cusp known to have endocarditis. However, the 2 sheep known to have no evidence of infection showed no evidence of CD8 positive cells (Fig. 4). The CD8 staining was in the proximity of the cell nuclei as represented by DAPI staining, as would be expected.

### Quantification of complement

**Fig. 5:**
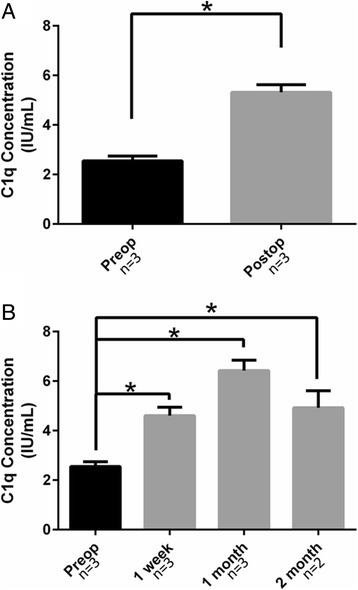
Quantification of complement in sheep serum pre and post valve-implantation- **a.** The serum C1q concentration preoperatively was 2.5 ± 0.2 IU/mL, which rose to an average of 5.3 ± 0.3 IU/mL after valve implantation (*p* = 0.00007). **b.** Serum C1q concentrations are shown at each postoperative time point. At 1 week, C1q concentrations were 4.6 ± 0.3 IU/mL (*p* = 0.0008, compared to preoperative concentrations) and reached their peak at 1 month (6.4 ± 0.4 IU/mL, *p* = 0.005). Two months postoperative C1q concentrations were 4.9 ± 0.7 IU/mL (*p* = 0.02). Oneweek and 1 month concentrations were also different from one another (*p* = 0.03)

Complement, specifically C1q, was measured in the serum of all 3 sheep at designated time points. There was a statistically significant difference (*p* = 0.00007) in the serum C1q concentration before valve implantation (2.5 ± 0.2 IU/mL) and at averaged time points after valve implantation (5.3 ± 0.3 IU/mL) (Fig. 5a). At 1 week, the concentration rose to 4.6 ± 0.3 IU/mL and reached its peak at 1 month (6.4 ± 0.4 IU/mL). There was a statistically significant difference between preoperative concentrations and concentrations at 1 week (*p* = 0.0008), 1 month (*p* = 0.005), and 2 months (*p* = 0.02); 1 week and 1 month concentrations were also significantly different (*p* = 0.03) (Fig. 5b).

## Discussion

Decellularized porcine valves implanted into a sheep model triggered a xenogeneic immune response in the sheep. Specifically, we saw a rise in the humoral immune system by means of IgM and IgG antibodies that could be measured in sheep serum and this seemed to peak at about the 1 week time point. As would be expected, IgM antibodies were earlier than IgG antibodies. The downstream effect of the humoral immune system was likely via the complement system as C1q concentrations in the sheep serum peaked at 1 month. T lymphocytes did not seem to play a role in this immune response as indicated by CD8 immunostaining.

Decellularized porcine valves have been previously implanted into pediatric patients with devastating results. Three of the 4 children died secondary to complications from the valve and the 4th was explanted. Incomplete decellularization was thought to have led to the failure secondary to immune reaction in these patients [14]. One hundred and three patients were implanted with decellularized porcine xenografts after extensive animal trials with much better short term results. There were two early short-term deaths and one reoperation was required secondary to pseudoaneurysm formation. Follow up was only 2 years in 9 patients in the reported manuscript with good hemodynamic results, thus the long term implications of potential antigenic complications is yet to be known [15]. Konertz et al. introduced the Matrix P decellularized xenograft for use in the pulmonary position and showed feasibility [16]. Dohmen et al. implanted pulmonary decellularized and re-endothelialized xenografts into 12 patients and at 4 years’ time had hemodynamic and survival results comparative to patients receiving pulmonary allografts [17]. However, two independent groups using the Matrix P report failure, reoperation, and deaths [18, 19]. Thus, large animal trials are essential to detailing potential immunogenic complications of these decellularized valves before proceeding with further human trials.

To our knowledge there have been no large animal trials detailing the immune response to a “xenotransplant” of a decellularized heart valve. However, Mosby et al. show that decellularization significantly reduced cellular and humoral immune response to allograft (not xenograft) tissue in a rat infra-renal implantation model [20]. Bloch et al. compare human serum levels of antibodies against α-gal in patients implanted with glutaraldehyde fixed porcine valves versus decellularized porcine valves and conclude that decellularized valves fare much better [21]. However, they do not examine antibodies against non α-gal surface antigens. Both of these studies are in line with our results in that decellularization is likely effective against α-gal antigens. However, we are the first to report a cross-species immune response to non α-gal antigens, the clinical relevance of which has yet to be demonstrated.

In the present study, decellularization with 1% SDS did seem to remove α-gal antigens from porcine valve conduits based on ELISA assay. Other decellularization methods have also been shown to remove α-gal antigens [8, 22] and we do not compare decellularization methods in this study. However, SDS is known to be the least harsh of the decellularization methods [23, 24] and damage of the extracellular matrix and resulting reduced durability of the valve must be weighed against antigen removal. Other methods of removing α-gal antigens beyond decellularization are also available, such as washing with α-galactosidase [25, 26].

Alpha-gal knockout animals have taught us that there are non-α-gal antigens that remain a barrier to the long term durability of xenografts. In vitro tests show the existence of non-gal antigens on decellularized heart valve tissue [27]. However, our results are the first in vivo experiments. Being a non-primate, the sheep model does not produce α-gal antibodies and thus is the ideal model to test for the existence of non-gal antigens on our decellularized porcine tissue. Sheep are quite similar to humans in terms of their immunological system. Specifically, humans and sheep are similar in the conservation of surface molecules on leukocytes, cytokines, complement cascade, and organization of the major histocompatibility complex [28]. There is likely not a large cohort of non-gal antibodies in circulation and production is ramped up only after exposure to a xenograft [29]. Our data support this as anti-sheep staining is far reduced on decellularized porcine tissue when incubated with preoperative sheep serum.

When examining the serum concentrations of anti-pig IgG and IgM at different postoperative time points, we found a sustained sheep IgG and IgM response in our decellularized porcine tissue with our immunohistochemistry. We do not have enough data to show the relationship between serum anti-pig antibodies and those that attack the valve. As such, the clinical impact of this antibody formation cannot be drawn from this study.

We found that complement, specifically C1q, is increased in sheep serum after implantation of decellularized porcine valves as early as 1 week. This implies that complement is involved in the immune response against decellularized valves. This is supported by solid organ transplantation literature [30]. Complement is also known to be activated by IgM and IgG antibodies which we show are also involved in the immune response [31]. Overall, it seems that decellularization does not effectively remove all antigens from porcine valves and as such the humoral immune system does react to implanted valves.

There does not seem to be a T-cell mediated immune response against decellularized porcine tissue in the 2 month time frame that we studied. Extrapolating from solid-organ transplantation literature, T-cells are a later response than antibody or complement mediated responses [32, 33]. In fully cellular organs, this may only mean a few days, but in a decellularized valve with low antigen burden, T cell response could likely be delayed for more than a few months. Longer time points are necessary to fully address the role of cytotoxic T cells in the immune response against decellularized porcine valves. However, in the first two months cytotoxic T cells seem to have a minimal role in the response against decellularized porcine tissue.

We have previously published our analysis of the implanted valves and reason for failure in vivo [12]. In short, one sheep received a valve sterilized with 1500 Gy gamma radiation and at explanation the valve was found to have endocarditis. This sheep’s valve was used as a positive control for the cytotoxic T-cell experiment in this study. The evaluation for anti-pig antibodies could have been affected as well and as such we used an ovine decellularized valve sterilized with the same dose of gamma irradiation for a control. This sheep’s serum was utilized in all other experiments as we did not believe infection would affect the results of those experiments. The other two sheep received valves with 3000 Gy gamma irradiation and were stenotic after 2 months in vivo. We believe the ultimate clinical fate of these valves was secondary to the damage induced by gamma irradiation and not by the antigenicity data we present in this study. Our data support the claim that decellularized porcine valves incite a humoral immune response in the sheep, but the effect of this immune response on the function of the valve is unknown at this time. Future in vivo studies must correlate immune response with the clinical function of the valve.

### Limitations

This study was performed using a sample size of *n* = 3 sheep. While the results were consistent between animals and the immune response was highly significant compared to control groups, we cannot completely rule out the possibility that other sheep may have a less significant response due to the low sample size studied. In addition, sheep do not produce α-gal antibodies, and it is consequently only possible to study the response to non-α-gal antigens using this model. Humans do produce α-gal antibodies and may therefore show an even stronger overall immune response to the decellularized tissue studied here. Nevertheless, it was the objective of this study to assess only the immune response to non- α-gal antigens. Finally, a sham surgery group was not used in this study and we therefore cannot completely separate the immune response caused by the cardiopulmonary bypass circuit from that caused by the decellularized tissue implant. However, the immune response remained strong well beyond when it otherwise would have been expected to diminish due to surgery alone, which strongly implicates the implant as the primary cause of the response observed.

## Conclusions

Decellularized porcine valves elicit an immune response when implanted into sheep. The immune response is antibody and complement mediated and does not seem to involve cytotoxic T-cells directly during the first 2 months. Furthermore, antigens other than α-gal appear to elicit the immune response against valves decellularized by the SDS method. The clinical impact of this level of antigenicity is presently unknown. However, the effect of immune response on valve durability must be carefully evaluated before moving into human studies.

## Additional file

Additional file 1: Methods Supplement (DOCX 19 kb)

## Abbreviations

ELISA: Enzyme-linked immunosorbent assay
PBS: Phosphate buffered saline
PBS-T: Phosphate buffered saline with tween-20
RIPA: Radio immunoprecipitation assay
SDS: Sodium dodecyl sulfate
α-gal: Galactose-alpha 1–3-galactose
