# Supplementary material for: Xenoantigenicity of porcine decellularized valves
Source: J Cardiothorac Surg. 2017 Jul 17;12:56. doi: 10.1186/s13019-017-0621-5 (PMC5514525; doi:10.1186/s13019-017-0621-5)
Supplement: Additional file 1: — Methods Supplement (DOCX 19 kb) [file 13019_2017_621_MOESM1_ESM.docx]

Additional file 1: Methods Supplement:

α-gal Epitope ELISA

An ELISA development kit was purchased from KPL (Gaithersburg, MD, product 54-62-18) which contains: coating solution concentrate, HRP-labeled Anti-Mouse IgG (H+L), ABTS Peroxidase Substrate System, ABTS Stop Solution. Human serum albumin (Sigma-Aldrich, St. Louis, MO, product A1653-5G) prepared in PBS at 1%, was used as a blocking agent. PBS (product 161-0780) was purchased from Bio-Rad (Hercules, CA). Tween-20 (product P1379-100ML) was purchased from Sigma-Aldrich, St. Louis, MO. Alpha Gal monoclonal antibody, M86 is from Enzo Life Sciences, Inc. (Farmingdale, NY, product ALX-801-090).

Protein extracts were obtained from fresh porcine aortic cusps (positive control, n=5), decellularized porcine aortic cusps (n=5), and human aortic cusps (negative control, n=3). The protein extracts were made by flash freezing the tissue in liquid nitrogen and homogenizing into a powder using a tissue mill. The powder was then added to 10 mL of RIPA lysis buffer with 1x protease inhibitor and vortexed for 10 mins at room temperature. Finally, the lysate was centrifuged at 12,000 RPM for 10 mins and the supernatant was used to coat 96-well polystyrene ELISA plates. Microtiter wells were coated with 1.5 - 3 µg of extract in coating buffer (100 µL of a 0.015 µg/µL solution) and allowed to absorb overnight at 4 ^o^C. Nonspecific binding was blocked by addition of 300 µL of 1% human serum albumin in PBS for 1 hr at room temperature (RT). Wells were washed 2x with wash buffer and 100 µL of α-Gal monoclonal antibody, diluted 50x in PBS, was added. The plate was incubated for 2 hr at RT and washed 4x with wash buffer. Secondary Ab-enzyme conjugate (goat anti-mouse IgG-HRP) was diluted to 0.5 µg/mL in PBS and 100 µL was added to each well followed by incubation for 1 hr at RT and 4x washes in a wash buffer. Substrate solution (100 µL) was applied for 15 min for color development and the reaction was stopped by the addition of 100 µL of stop buffer. Absorbance was determined at 405 nm and background absorbance was subtracted.

Animals and Operative Model

Juvenile sheep (n=3) 3 to 4 months of age and 30 to 40 kg of weight underwent a pulmonary valve replacement with decellularized and sterilized porcine aortic valves. All animals received humane care in compliance with the Principles of Laboratory Animal Care formulated by the National Society for Medical Research and the Guide for the Care and Use of Laboratory Animals. The study protocol was approved by the Institutional Animal Care and Use Committee at Mayo Clinic. The operations were performed through a left thoracotomy utilizing the fourth intercostal space to gain exposure. After heparinization, normothermic cardiopulmonary bypass was utilized to empty the heart, but the heart was not arrested. The main pulmonary artery was incised 1 cm above the right ventricular outflow tract and just below the bifurcation of the pulmonary artery. The native pulmonary valve cusps were excised. The decellularized heart valve was interposed in the pulmonary position with running sutures of 4-0 polypropylene proximally and distally. Intercostal nerve blocks were performed with 0.5% bupivacaine and epinephrine mixture prior to closure of the thoracotomy. Ceftiofur 5 mg/kg was given intramuscularly the day before the surgery and was repeated on the 3rd postoperative day. Cefazolin 50 mg/kg was given intravenously 15 mins prior to incision. Animals were kept indoors for the first week postoperatively and given 25 U/kg Heparin twice a day for 2 days postoperatively. Vital signs were monitored daily for one week.

Serum for immune testing

Ten mL of blood was drawn into clot tubes from each sheep (n=3) preoperatively, 1 week postoperatively, 1 month postoperatively and 2 months postoperatively (with the exception of our first sheep which was sacrificed at 1 month). The blood was centrifuged immediately at 3000 RPM for 10 mins. The top layer was stored at -80 °C for later analysis.

Anti-pig antibody staining

Fresh frozen blocks of 3000 Gy gamma irradiated (n=4) decellularized porcine aortic valve cusps were cut into 5 μm sections. Each section was incubated with a 1:25 dilution of sheep serum (obtained at different time points as described above) in 4 °C for 18 hrs. After 3 washes with PBS containing 0.1% Triton X, the sections were incubated with a 1:200 dilution of both rabbit anti-sheep IgM:FITC (AbD Serotec) and donkey anti-sheep IgG:Alexa Fluor 594 (Life Technologies) for 1 hr at room temperature in the dark. After secondary antibody incubation, ProLong Gold Antifade with DAPI (Life Technologies) was utilized for preservation and counterstaining. Our negative staining controls utilized PBS rather than sheep serum. All time-points of the collected sheep serum were tested: preoperative, 1 week postoperative, 1 month postoperative and 2 months postoperative. All images were taken with a confocal microscope with 20x magnification and utilizing the preoperative serum section for gain and pinhole control.

Anti-pig antibody ELISA

Microtiter wells (Thermo Scientific, Waltham, MA) were coated with 100 µL of a 10 µg/mL extract of decellularized porcine valves prepared in coating buffer in all wells except the calibrator curve wells. The calibrator curve wells were coated with 100 µL of a serial dilution of either IgG or IgM antibodies prepared in coating buffer (Kirkegaard & Perry Laboratories, Inc., Gaithersburg, MD) at 12, 6, 3, 1.5 µg/mL. Separate wells were used for each isotype. The plate was incubated at 4 ^o^C overnight to allow adsorption. The plate was washed 3x in 300 µL of PBS-T (PBS with 0.02% Tween-20) and 1% human serum albumin (Sigma-Aldrich, St. Louis, MO) in PBS was added as a blocking agent. The plate was incubated at room temperature for 1 hr, and then washed 2x in PBS-T. Sheep sera from the 3 sheep implanted with decellularized valves were added to the antigen-coated wells (100 µL of a 25 fold dilution in PBS), whereas PBS was added to the calibrator curve wells. A pair of antigen-coated wells received only PBS to monitor background. The plate was incubated for 2 hr at room temperature, and then washed 4x in PBS-T. A mixture (100 µL of a 3 µg/mL solution of each) of FITC conjugated anti-IgM (Bio-Rad, Hercules, CA) and Alexaflour 568 conjugated anti-IgG (Life Technologies, Grand Island, NY ) was added. The plate was incubated for 1 hr at room temperature and then washed 4x in PBS-T. PBS was added to each well (100 µL/well) and the fluorescence was measured using the maximum excitation and emission wavelengths for each fluorophore. Background fluorescence was subtracted and the calibration curves were fitted linearly. Standards and samples were assayed in duplicate in three independent ELISAs. Purified sheep antibodies from Fitzgerald Industries (Acton, MA), IgG (product 31R-1050) and IgM (product 31C-CH1313), were used to produce standard curves.

Cytotoxic T-lymphocyte staining

A portion of each cusp of the explanted valves (n=3) was placed in OCT and frozen. These blocks were then cut into 5 μm sections. Each section was blocked with 1% bovine serum albumin for 1 hr at room temperature. Anti-CD8 antibody (Abcam) was used at 1:100 dilution at 4 °C for 18 hrs. Goat anti-rabbit alexa fluor 488 (Life Technologies) was utilized at 1:200 dilution at room temperature for 1 hr. After secondary antibody incubation, ProLong Gold Antifade with DAPI (Life Technologies) was utilized for preservation and counterstaining. Rabbit IgG (1:100 dilution, Invitrogen) was utilized as the isotype control for each sheep section. The cusp of the sheep known to have endocarditis on the valve (previously published results) was utilized as a positive control and the other two sheep with no evidence of infection were used to determine the result. All images were taken with a confocal microscope with 20x magnification and utilizing the positive control for gain and pinhole control.

Quantification of Complement

Sheep C1q ELISA kit was purchased from BioTang and utilized to measure the pre and postoperative sheep serum samples of the 3 sheep implanted with decellularized valves. Samples were diluted 1:40 with the provided sample buffer. Standards and samples were run in technical triplicates and the experiment was repeated twice.
